# Implementation of the WHO Surgical Safety Checklist in an Ethiopian Referral Hospital

**DOI:** 10.1186/1754-9493-8-16

**Published:** 2014-03-28

**Authors:** Tom Bashford, Sophie Reshamwalla, Jacqueline McAuley, Nikole H Allen, Zahirah McNatt, Yohannes D Gebremedhen

**Affiliations:** 1 Division of Anaesthesia, Addenbrooke’s Hospital, University of Cambridge, Cambridge CB2 0QQ, UK; 2 Lifebox Foundation, London, UK; 3 School of Nursing & Midwifery, Glasgow Caledonian University, Glasgow, UK; 4 Global Health Leadership Institute, Yale University, New Haven, Connecticut, USA; 5 Department of Plastic and Reconstructive Surgery, Yekatit 12 Hospital, Addis Ababa, Ethiopia

**Keywords:** Checklists, Anaesthesia, Surgery, Ethiopia, Patient safety, Low-resource settings, Quality improvement

## Abstract

****Background**:**

The WHO Surgical Safety Checklist has a growing evidence base to support its role in improving perioperative safety, although its impact is likely to be directly related to the effectiveness of its implementation. There remains a paucity of documented experience from low-resource settings on Checklist implementation approaches. We report an implementation strategy in a public referral hospital in Addis Ababa, Ethiopia, based on consultation, local leadership, formal introduction, and supported supervision with subsequent audit and feedback.

****Methods**:**

Planning, implementation and assessment took place from December 2011 to December 2012. The planning phase, from December 2011 until April 2012, involved a multidisciplinary consultative approach using local leaders, volunteer clinicians, and staff from non-governmental organisations, to draw up a locally agreed and appropriate Checklist. Implementation in April 2012 involved formal teaching and discussion, simulation sessions and role play, with supportive supervision following implementation. Assessment was performed using completed Checklist analysis and staff satisfaction questionnaires at one month and further Checklist analysis combined with semi-structured interviews in December 2012.

****Results and discussion**:**

Checklist compliance rates were 83% for general anaesthetics at one month after implementation, with an overall compliance rate of 65% at eight months. There was a decrease in Checklist compliance over the period of the study to less than 20% by the end of the study period. The ‘Sign out’ section was reported as being the most difficult section of the Checklist to complete, and was missed completely in 21% of cases. The most commonly missed single item was the team introduction at the start of each case. However, we report high staff satisfaction with the Checklist and enthusiasm for its continued use.

****Conclusion**:**

We report a detailed implementation strategy for introducing the WHO Surgical Safety Checklist to a low-resource setting. We show that this approach can lead to high completion rates and high staff satisfaction, albeit with a drop in completion rates over time. We argue that maximal benefit of the Surgical Safety Checklist is likely to be when it engenders a conversation around patient safety within a department, and when there is local ownership of this process.

## Background

Ethiopia is one of the world’s poorest countries with a population of 82.8 million yet only 0.2 physicians per 10,000 people [1]. However, it is currently undergoing a period of extensive development of its healthcare services, with a rapid expansion of healthcare professional training and a substantial investment in healthcare facilities. Although data on surgical outcomes is limited, published figures from the largest referral hospital in Ethiopia show an all-cause surgical mortality of 7% [2]. The Clinton Health Access Initiative (CHAI) and the Yale Global Health Leadership Institute (GHLI) are working together with the Ethiopian Federal Ministry of Health as the Ethiopian Hospital Management Initiative (EHMI) to improve healthcare services across Ethiopia through the introduction of the 2010 Ethiopian Hospital Reform Implementation Guidelines (EHRIG) [3].

The World Health Organisation (WHO) Surgical Safety Checklist has been shown to reduce surgical mortality in a range of settings [4] and its introduction to Ethiopian hospitals is an integral part of the EHRIG. In addition, the Checklist is endorsed in both the 2009 WHO Safe Surgery Guidelines, [5] and in the 2010 International Standards for a Safe Practice of Anaesthesia, [6] and has a robust evidence base supporting its positive effect on surgical mortality and morbidity [7]. While critics point out that Checklists alone are not sufficient to improve patient safety, and must be accompanied by wider strategies for quality improvement, [8] it is hoped that implementation of the WHO Surgical Safety Checklist as part of the wider EHRIG reforms will reduce surgical mortality and morbidity in Ethiopia.

We report the introduction of the WHO Surgical Safety Checklist to the plastic & reconstructive surgery department of Yekatit 12 Hospital, a referral hospital in Addis Ababa, as the result of a collaborative project between the hospital, CHAI, Yale GHLI, clinical health volunteers from Voluntary Service Overseas (VSO), and Lifebox Foundation. Our aim is to demonstrate how the Checklist may be implemented in Ethiopia, building on those strategies developed from experience in both high and low-resource settings, and provide further lessons for other low-resource centres.

## Methods

### Selection of site, funding and ethical considerations

The project took place between December 2011 and December 2012. Yekatit 12 Hospital was initially identified as the implementation site via two pathways. There was a locally identified need for an improvement in anaesthetic and surgical safety checks with the WHO Surgical Safety Checklist chosen by the plastic & reconstructive surgery department as one option to achieve this. In addition, CHAI and Yale GHLI had selected Yekatit 12 Hospital due to the existing placement of a Voluntary Service Overseas (VSO) anaesthesiologist (TB) at the hospital to act as a liaison between the various institutions. The plastic & reconstructive surgery department was the ideal setting for introduction of the Checklist due to its history of innovation, smaller pool of staff, and lack of emergency caseload. Implementation followed a four-step process: planning and consultation between December 2011 and April 2012; formal training at the start of April 2012; supportive supervision; and evaluation at one month and again in December 2012. The production of video teaching aids to help dissemination of the project was completed between the formal training and the final evaluation.

VSO volunteers (TB, JM) were placed at the hospital for the period spanning planning, training, supportive supervision and the initial evaluation phase, as part of previously existing long-term volunteer placements. CHAI/GHLI staff (ZM, NHA) were able to support the project as part of their ongoing role within the EHMI. Lifebox provided advice remotely in the initial phase but provided human resources (SR) to help with the long-term evaluation in conjunction with CHAI/GHLI (NHA). Funding for the project beyond staffing costs came from VSO, who supported the costs of the training day, and CHAI/GHLI who provided administration and printing costs, *per diem* costs for staff attending training, costs for CD-ROM production and filming costs.

Yekatit 12 Hospital did not, at the time of planning, have an institutional policy regarding quality improvement processes and ethical review. The local lead for the project (YDG) was consulted and advised that the introduction of agreed national initiatives would not require explicit ethical approval from the Regional Health Bureau (RHB), the local responsible body. Use of the Checklist is clearly promoted within the EHRIG [3] and as such is endorsed by both the RHB and the Ethiopian Federal Ministry of Health. In addition, the full support of the Medical Director of the hospital was obtained for implementation, analysis and publication. It was decided that identification of the site of the project was important in order to be able to publicise the implementation throughout Ethiopia. However it was decided that staff would not be identified by name, and that it would be entirely optional to participate in feedback. All staff who provided feedback were made aware that anonymous, aggregated responses would be published and that free text comments would be attributed to roles but not named individuals. Furthermore, it was explained that returning of a completed questionnaire was considered consent for this data to be published but that returning questionnaires was optional. As the implementation involved the adoption of recommended national guidelines, local staff were not able to refuse to participate in the implementation itself.

The need for ethical review in quality improvement is a complex area. Key important points include: the proposed change was in accordance with existing national and international guidelines; the proposed change would affect all patients equally; the proposed change would result in the optimum delivery of care but would not change the nature of that care; that patients’ rights would be respected at all times. When considering the publication of the work, it was felt that this represented experience sharing rather than generalizable scientific knowledge and that no further ethical review was necessary before publication.

### Planning and consultation

**Figure 1:**
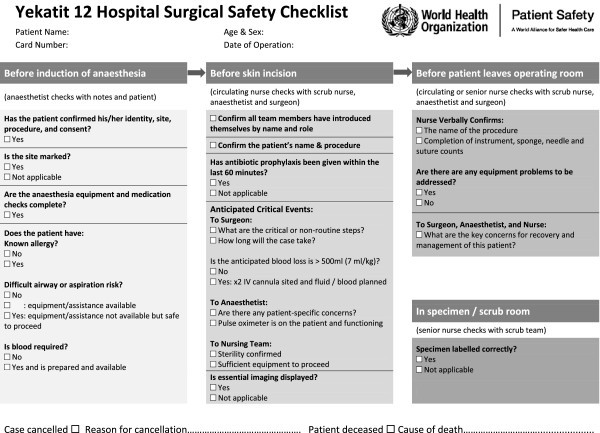
The Yekatit 12 Hospital Surgical Safety Checklist.

The initial phase involved a five-month consultation period, beginning in December 2011, with local surgical, nursing, anaesthetic, and ward staff. This began with informal discussions among the team during normal working hours, discussing the nature of a Checklist approach and potential benefits to patients. A draft Checklist was produced and was then circulated to anaesthetic, nursing and surgical teams for private review and comment. Comments were collated by team leaders and a revised Checklist was produced and circulated to all staff. A plenary meeting was then chaired by the department lead (YDG) to which all staff were invited and at which all specialities were represented. This meeting finalised the Checklist to the satisfaction of all teams and a final version was produced (Figure 1). During this time the project was discussed with the Medical Director of the hospital and once approval was obtained a funding application was submitted to VSO to help finance the implementation. Preparations were made by CHAI/GHLI to produce paper Checklists, and wall chart versions of the Checklist were prepared to be used in the relevant clinical areas. The teaching materials were produced, the follow-up questionnaire designed, and arrangements made for the formal training day. This required identifying a day during which theatres had a scheduled closure, so that implementation would not impact on patient care, in addition to arranging a teaching room and catering.

### Training

A programme combining formal teaching and supervised simulation was prepared by CHAI, GHLI, VSO staff and local surgeons. Formal teaching involved a single half-day workshop session for all staff using WHO video material on the origins and use of the Surgical Safety Checklist. This was led by one of the VSO volunteers (TB), chaired by the local lead (YDG) and supported by CHAI/GHLI and VSO (ZM, JM). Staff were provided with a CD-ROM containing background information on the Checklist and encouraged to review this in their own time. Written resources were also on display in the surgical department. Following the half-day workshop was a half-day simulation and role play session in the operating theatre run by VSO volunteers (TB, JM). During simulation, staff were given the opportunity to use the Checklist in their normal roles, while in the role play session they were encouraged to adopt the role of a different member of the surgical team. Although training took place during normal working hours, staff were provided with a small inducement of 100 ETB (approx. £3.50) to attend. As English and Amharic are used interchangeably, the Checklist was provided in both languages for all staff.

### Supportive supervision

Batches of the adapted paper Checklists were copied and placed in each theatre, with VSO volunteers (TB, JM) supporting the theatre staff in completing the Checklist for the first two weeks. This involved attending cases while observing and supervising use of the Checklist until it became routine practice. VSO staff usually took the role of “observer” and did not take responsibility for reading or completing the Checklist unless also directly involved with patient care. The focus of the supervision was to remind and encourage staff to use the Checklist, and to troubleshoot any problems that arose.

### Evaluation

Implementation was assessed at one month and eight months after training. At one month, two outcome measures were chosen: the percentage of general anaesthesia cases for which a Checklist was successfully filled out (compliance), and staff satisfaction. Compliance was calculated by comparing the number of completed Checklists with the number of general anaesthesia cases recorded in theatre log books, and staff satisfaction was assessed using a questionnaire distributed by VSO volunteers (Additional file 1).

The calculated compliance was communicated to staff before completion of the questionnaire to inform responses. The staff questionnaire was designed to evaluate three key areas: the introduction of the Checklist to the department; the Checklist itself; and the experience of using the Checklist one month after introduction. The questionnaire was prepared in English and employed both Likert scale questions along with free text boxes for further comments. Limited time and resources prevented the production of a formally validated questionnaire.

Twenty questionnaires were prepared to mirror the twenty participants at the training workshop however it was expected that some of the responses would be from staff who could not attend the initial training. Due to staffing levels, only nineteen of these were distributed during the period of evaluation. Results, including free text, were transcribed verbatim and entered into Microsoft Excel© (Microsoft Corporation, Washington, USA) for analysis. The results of the staff questionnaire were then communicated informally to the team.

A more detailed evaluation was conducted by CHAI/GHLI and Lifebox Foundation (NHA, SR) in December 2012 to assess Checklist compliance and identify lessons to aid future implementations. This evaluation consisted of calculating monthly compliance and assessing the overall completeness of each Checklist through a retrospective analysis of theatre logbooks and the Checklist documentation kept in the operating theatre. Semi-structured interviews with members of nursing, surgical and anaesthetic teams were undertaken using specific questions (Table 1) but encouraging free discussion. The results of this follow-up evaluation were then given to senior members of the theatre team to disseminate to colleagues.

**Table 1:** Semi-structured interview questions at 8-month follow-up

**Questions**	
How do you find the checklist? (open, introductory question)	
Do you find the checklist easy or difficult to use?	
Why do you think you are using the checklist less now compared to when you first started?	
Specifically, what do you think about:	
1. Compliance rate?	
2. Sign out?	
3. Team introductions?	
4. Confirmation of name and procedure?	
5. Counting instruments and sponges?	
What are the next steps for the checklist at Yekatit 12?	

All interviewees were asked the five specific questions. Generic questions were used to facilitate discussion around the points raised.

## Results and discussion

### Checklist development

The Yekatit 12 Hospital Surgical Safety Checklist (Figure 1) shows specific changes to the template available from the WHO. Key features required by local staff were: the inclusion of patient data on the Checklist form; the provision of a separate ‘In specimen /scrub room’ section; the movement of equipment checks and IV access questions to the ‘Time out’ section as the ‘Sign in’ occurs in a small ante room before the patient has walked into the operating theatre and been attached to monitoring equipment; the clear prescription of who is to lead each section; and the question ‘Is there sufficient equipment to proceed?’ in the ‘Time out’ section. In addition, it was felt that there should be no place for a staff member to sign or date the form as it was felt this would reduce compliance: staff reported a nervousness that should they sign the form and some mistake be found, that they would then be held accountable for any harm that arose. While minor, these changes were felt to be essential to the Checklist being accepted by the whole theatre team.

### One-month evaluation

#### Compliance

Compliance at one month was 83% (38/46 general anaesthesia cases) with only 6/38 (16%) Checklists incompletely filled out. The six incomplete Checklists had completed ‘Sign in’ and ‘Time out’ sections but did not have a completed ‘Sign out’ component.

#### Staff satisfaction questionnaires

Nineteen staff questionnaires were distributed with a response rate of 84% (16/19). The distribution of respondents is displayed in Table 2. Seven respondents had not attended the initial training and were unable to answer the questions regarding reflection on the training day.

**Table 2:** Workshop attendees and one-month evaluation questionnaire respondents

**Respondent**	**Attended training workshop (*n*** **= 20)**	**Questionnaires returned (max 19)**
Surgeons	6	3
Theatre nurses	8	3
Surgical residents	0	4
Anaesthetists	4	1
Not specified/other	2	5
**Total**	**20**	**16**

Values are number. ‘Not specified/other’ refers to those not fulfilling one of the identified categories, and respondents who did not identify their profession on their returned questionnaire. Three nurses accepted questionnaires but later declined to respond due to perceived difficulties with language.

#### *‘Introduction of the WHO Surgical Safety Checklist*’

Of the 16 respondents, 12 (75%) felt that there was a need to improve surgical safety before the introduction of the Checklist, while 11 (69%) felt there was a need to improve communication. Five had been aware of the existence of the WHO Surgical Safety Checklist before its introduction. Thirteen (81%) felt that introduction of the Checklist had been successful and that they would be confident introducing it to a new colleague joining the department.

Nine staff had attended the initial training day and were able to comment on their satisfaction with the training methods. All nine agreed that the training components of importance were: the WHO videos of ‘how to’ and ‘how not to’ use the Checklist; the simulation and role play session in the operating theatre; and the support after the training day. Five felt that the morning workshop had been an important tool, while four agreed that both the informal discussion period and plenary staff meeting to agree Checklist modifications had been important. Of note, the three respondents who identified themselves as nurses all answered ‘Disagree’ to the questions regarding their ability to contribute before implementation: their ability to give their opinion on the Checklist before its introduction; the importance of both the informal discussion period and the plenary staff meeting; and importance of the morning workshop (Additional file 1).

#### ‘Your opinion on the WHO Surgical Safety Checklist’

100% of respondents responded that the Checklist improved staff communication, patient safety and overall patient care. 94% felt that it was easy and quick to complete, and had improved relationships between theatre staff. Responses to the issue of cancellations and equipment problems were mixed, with 75% responding that using the Checklist reduced the number of cancellations, and 81% agreeing that the use of the Checklist allowed equipment problems to be addressed. All respondents agreed that they would recommend using the Checklist to a colleague, and would like staff to be using the Checklist were they themselves to be undergoing an operation.

#### The WHO Surgical Safety Checklist after one month

Only six respondents felt that a completion rate of 83% for general anaesthesia cases was acceptable, with all respondents agreeing that the Checklist should be used in all general anaesthesia cases. Despite this, 15 felt that it was working well within the department, with 11 also stating that the use of the Checklist in the department could be improved. While 12 respondents felt that the Checklist should be used for all spinal anaesthesia cases, only 7 felt that it should be used for local anaesthesia/sedation cases and only 4 felt it was applicable to day cases.

The sections of the questionnaire focusing on which parts of the Checklist were most difficult, and which parts were most important, were poorly completed. While all respondents completed the section fully, many checked the ‘Agree’ or ‘Disagree’ box for all questions despite the instructions on the questionnaire [Additional file 1], making interpretation difficult. Taking the total of all ‘Agree’ answers for each statement as a measure of agreement, the ‘Sign out’ section was seen as the most difficult to complete with eight ‘Agree’ responses. In contrast the ‘Sign in’ and ‘Time out’ sections had four ‘Agree’ responses each and the ‘Specimen labelling’ section had no ‘Agree’ responses. Using the same method of assessment, the ‘Sign in’ section was classified as the most important with 12 ‘Agree’ responses, as compared to 10, 7 and 6 for the ‘Time out’, ‘Sign out’ and ‘Specimen labelling’ sections respectively.

#### Free text comments

The staff were invited to express their feelings about the WHO Surgical Safety Checklist in a free-text section at the end of the questionnaire. Eleven of the sixteen respondents chose to offer comments, which ranged from single-line answers to extended discussions. Many staff noted how easy the Checklist is to complete and how little an administrative burden it imposes. The following quotes are taken from returned questionnaires:

“It is nice, easy to use.” - *Scrub Nurse*

“It is so easy, not time consuming and very helpful.” - *Health Officer*

“It is comprehensive and easily understandable.” - *Surgical Resident*

The responses also revealed confidence among staff in the Checklist’s ability to improve patient safety and overall patient care:

“It is a good format that helps for the ultimate safety of the patient in the OR [operating room].” - *Surgeon*

“I found it is very important for patient care.” - *Surgical Resident*

“The SSCL [Surgical Safety Checklist] in the OR has made a difference …for patient safety as well as for smooth working environment.” - *Surgical Resident*

“Improves pt [sic] safety, decreases cancellations, improves pt [sic] care, address equipment problems.” – *Unknown*

“I have seen in the ward that patients now are not getting infections, before there were so many, the WHO SSCL [Surgical Safety Checklist] is so good, patients get their antibiotics properly…” *- Health Officer*

A desire for wider dissemination throughout the country was also reflected:

“The model at Y12 [Yekatit 12 Hospital] must be applied to other hospitals.” *- Scrub Nurse*

“I would like to recommend just to extend this training to other hospitals.” - *Health Officer*

“I request you to promote and implement in other centres.” - *Surgical Resident*

Some staff suggested ideas regarding future use of the Checklist at Yekatit 12:

“Have regular meeting to improve and continue using SSCL [Surgical Safety Checklist]” – *Unknown*

“Repeat training for newcomers, regular monitoring and supervision.” – *Surgeon*

### Eight-month evaluation

#### Completion and compliance rates

**Figure 2:**
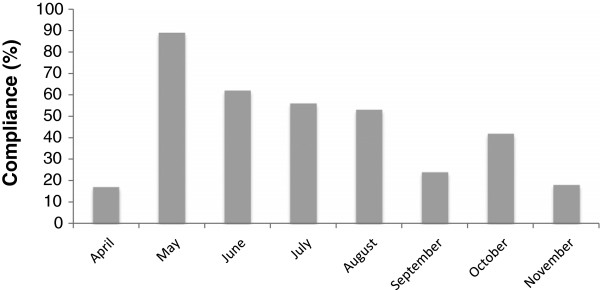
Checklist compliance over the eight months since implementation.

**Figure 3:**
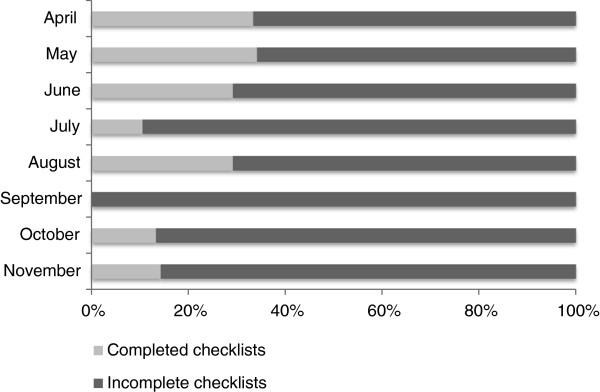
Checklist completion by month after implementation.

A total of 289 (general anaesthetic) operations were carried out between 24th April and 1st December 2012 according to theatre logbooks. A total of 187 (65%) Checklists were located. 40 (21%) Checklists were “complete” meaning that every item on the Checklist had been checked or commented on. Compliance and completeness figures for the Checklist for the eight months after implementation are given in Table 3, with the exclusion of 46 Checklists for which a date was not entered. Compliance with the Checklist decreased over time with the highest compliance rate seen soon after the training. The lowest compliance rate was seen in November 2012, with 7 (18%) operations having Checklists completed (Figure 2). The lowest number of fully completed Checklists was collected for September 2012 (Figure 3).

**Table 3:** Month-by-month analysis of completed checklists at eight-month evaluation

**Month**	**Total number of checklists**	**Completed checklists**	**Incomplete checklists**	**Number of GA cases logged**	**Compliance rate**	**Completeness rate**
April	3	1	2	18	17%	33%
May	41	14	27	46	89%	34%
June	24	7	17	39	62%	29%
July	19	2	17	34	56%	11%
August	24	7	17	45	53%	29%
September	8	0	8	33	24%	0%
October	15	2	13	36	42%	13%
November	7	1	6	38	18%	14%
**Totals**	**141**	**34**	**107**	**289**	**NA**	**NA**
**Mean**	**NA**	**NA**	**NA**	**NA**	**45%**	**20%**

Note: 46 checklists (6 complete, 40 incomplete) did not have the date entered and have not been analysed.

#### Item-by-item analysis

**Figure 4:**
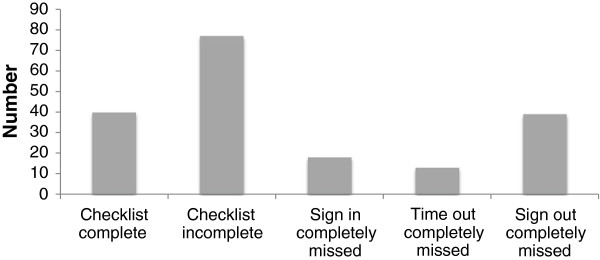
Breakdown of omitted Checlist items by section over the eight months since implementation.

The Yekatit 12 Checklist contains 22 Items to be checked. One-hundred and eighty seven Checklists were individually examined and a total of 4,114 checks were analysed to find out which items were most commonly missed. Overall there were 669 missed checks. The sign‒in was completely missed 18 times (9%). The time-out and sign‒out were missed 13 (7%) and 39 (21%) times respectively (Figure 4). The single check that was most frequently missed was item 7: “Confirm all team members have introduced themselves by name and role”. The second most frequently missed check was item 8: “Confirm the patient’s name & procedure”. The third most frequently missed check was item 22: “Specimen labelled correctly”. The frequency of missing items in partially completed Checklists is given in Table 4.

**Table 4:** Missing items in checklists over the eight months analysed

**Item no.**	**Checklist item (Yekatit 12 checklist)**	**Number of times missing**	**%**
	**Sign in**		
**1**	Has the patient confirmed his/her identity, site, procedure and consent?	34	5
**2**	Is the site marked?	21	3
**3**	Are the anaesthesia equipment and medication checks complete?	18	3
**4**	Does the patient have a known allergy?	21	3
**5**	Does the patient have a difficult airway or aspiration risk?	19	3
**6**	Is blood required?	21	3
	**Subtotal**	**134**	**20**
	**Time out**		
**7**	Confirm all tem members have introduced themselves by name and role	77	12
**8**	Confirm the patient’s name and procedure	71	11
**9**	Has antibiotic prophylaxis been given within the last 60 minutes?	17	3
	***Anticipated critical events to surgeon:***		
**10**	What are the critical or non-routine steps	13	2
**11**	How long will the case take?	13	2
**12**	Is the anticipated blood loss > 500ls?	20	3
	***Anticipated critical events to anaesthetist:***		
**13**	Are there any patient-specific concerns?	17	3
**14**	Pulse oximeter is on the patient and functioning?	15	2
	***Anticipated critical events to nursing team:***		
**15**	Sterility confirmed	14	2
**15**	Sufficient equipment to proceed	14	2
**17**	Is essential imaging displayed	37	6
	**Subtotal**	**308**	**46**
	**Sign out**		
**18**	Nurse verbally confirms name of procedure	41	6
**19**	Completion of instrument, sponge, needle and suture counts	41	6
**20**	Are there any equipment problems to be addressed?	40	6
**21**	What are the key concerns for recovery and management of this patient?	41	6
**22**	Specimen labeled correctly	64	10
	**Subtotal**	**227**	**34**
	**Total**	**669**	**100**

Percentages are given to the nearest whole number.

#### Semi-structured staff interviews

Seven staff were interviewed; three theatre nurses, two surgeons and two anaesthetists. Responses are grouped according to the five structured questions (Table 1).

1. Compliance Rate

During implementation it had been agreed that the Checklist would only be used for major cases requiring a general anaesthetic. Cases requiring a local anaesthetic or short cases (less than one hour) requiring ketamine sedation did not require a Checklist to be completed despite often being recorded in the theatre logbook as a general anaesthetic case. In addition staff commented that on occasion, the completed Checklists had been placed in the patient notes rather than in the identified folder. Staff felt that both of these factors may have led to an underestimation of compliance rates.

2. Sign-Out

The staff acknowledged that the sign out was difficult to remember to do in the busy theatre environment and often remembered to do it only after the patient had left the operating room. They also commented that the lack of a dedicated person to lead the sign out may contribute to it being forgotten. In contrast they felt that the sign-in being led by the anaesthetist, and the time-out involving the entire team, helped make these sections easier to complete.

3. Introductions

Confirming the team members by name and role was the most missed check. The explanation given for this was that a lack of staff turnover and degree of familiarity with each other made this check appear less important.

4. Confirmation of the patient’s name and procedure

This was the second most frequently missed check on the Yekatit 12 Hospital Checklist. Staff explained that this was a result of common discrepancies between the planned operation and that eventually performed (e.g. a burn patient who is scheduled for a skin graft may end up requiring a debridement). They explained that they often documented the procedure that the patient had undergone on the Checklist in response to item 18: “Nurse verbally confirms name of procedure”. They reported a reluctance to check the procedure before the start of the case in order to prevent a discrepancy in documentation should the final procedure differ from that planned.

5. Counting of sponges and instruments

Staff reported that there was no habit for counting swabs and instruments in the plastics unit at Yekatit 12, despite the analysis showing that when the sign out was done, this question was rarely missed. Further inquiry revealed that instead of physically counting the swabs and instruments, a visual check of the wound or cavity was done by the surgeon and communicated to the person completing the Checklist. Their explanation was that the cavities are very small in plastic surgery, or the surgery is superficial, therefore counting instruments and sponges was not felt necessary. The expectation reported by staff at Yekatit 12 was that counting swabs and instruments was only relevant to abdominal surgery. They noted however that counting packs during some procedures (such as palatoplasty) was important and reported that when these procedures were performed the swab count was performed and checked explicitly as part of the ‘Sign out’.

## Discussion

Introducing the WHO Surgical Safety Checklist to the clinical environment can be challenging. Published experience from high-resource settings has identified a number of strategies to aid the introduction of the Checklist into routine clinical practice [9-11]. These emphasise a supported implementation process with time taken to enlist local leaders, educate staff in the benefits of adopting the Checklist, deliver formal training, and repeatedly reinforce Checklist use during the initial phase. Applying these lessons to low-resource settings is difficult, where training budgets and facilities, institutional hierarchies, organisational leadership, and clinical workload can all interfere with quality improvement initiatives. In addition, there are limited reports of Checklist implementation in low-resource settings [12-14]. Yuan et al. [13] report the effects of a successful implementation in Liberia with a significant improvement in surgical processes and outcomes, but with variations seen at the institutional level suggesting that the process, and outcomes, of implementation are highly context-dependent [13]. An earlier cohort study by van Klei et al. [12] supports the intuitively reasonable idea that Checklist effectiveness will be directly linked to the effectiveness of implementation [15]. Rowe et al. [16] highlight the relative effectiveness of a combined managerial and educational approach using group processes, supervision, audit and feedback to improve quality in low resource settings, and this appears to be borne out in experience to date of Checklist implementation in these settings [16].

Our results demonstrate that an implementation strategy based on consultation, training, and support, can lead to high initial Checklist compliance (83%) and completeness (84%) rates in addition to high levels of staff satisfaction. Simulation sessions and role play were received well with the majority of staff commenting that this was an adequate method of training. We believe this helps break down barriers to communication and fosters a sense of team working. We note the encouraging ideas for future use of the Checklist from within the department, including repeating the training for new staff and continued modification of the Checklist. Expansion of Checklist use to other areas of the patient pathway may lead to even greater improvements in surgical mortality and morbidity [17].

Both compliance and completeness rates decreased in the months following implementation, despite the ongoing presence of VSO staff (TB, JM) and local leaders (YDG). This mirrors experience from other settings and can be due to a number of factors; a perception that the improvement has been made and that continued effort is no longer required; staff turnover; or loss of local leadership. In low-resource settings there may also be more material reasons, for example a temporary lack of either paper or printing facilities to create new copies of the Checklist, or sometimes even a lack of a pen to complete it. It was beyond the scope, and remit, of this project to measure outcomes beyond those reported, however it would have been instructive to monitor how changes in team behaviour, communication, safety processes and patient outcome varied with Checklist compliance over time.

Checklist completion and compliance may not represent an accurate reflection of practice. Completion of a paper Checklist does not guarantee that the expected steps have been followed. More optimistically, lack of completion of a paper Checklist does not prove that the behaviours enshrined in the Checklist have been ignored. Checklists can be quickly run through verbally by team members who are familiar with them, and it is not clear that this undermines their usefulness, although it seems reasonable to assume that the fidelity of the Checklist will decrease over time in this scenario. An ideal model of Checklist implementation might involve continued occasional visits by an external observer; this would allow assessment of Checklist use and reporting of any discrepancies between the recorded Checklist use and daily practice. In addition, a visiting observer would be able to provide continued coaching to staff to reinforce and develop Checklist use. It is important to note that this visiting observer need not be a ‘Checklist expert’ nor an overseas volunteer; neighbouring departments or institutions may be the best placed to support each other in their Checklist use, allowing sharing of common problems and solutions.

This study is an observational account of experience in a single centre. The centre was chosen as a site for implementation due to its strong local leadership, history of innovation and lack of emergency workload. As such it is not representative of the majority of operating sites in Ethiopia. In addition, longer-term follow-up is required to demonstrate a tangible improvement in patient safety through indices such as mortality, length of hospital stay, or post-operative infection rates. Importantly, post-implementation questionnaires were not formally validated so represent feedback rather than robust qualitative research. There is a reasonable possibility of reporter bias in the questionnaire feedback; despite the high response rate, the small numbers involved and the intimacy of the working environment mean that enthusiastic responses may reflect a desire to please friends and colleagues who have been working to implement the Checklist rather than reflecting deeply held opinions.

The strength of this work, however, is that it demonstrates implementation of the WHO Surgical Safety Checklist in a publically-funded institution in a low-resource setting using local leadership albeit in addition to long-term clinical volunteers and non-governmental organisations. This has allowed for not only the tailoring of the Checklist to the local context, but also for the post-training supervision. With their work on implementing the EHRIG, CHAI and Yale GHLI are well placed to assess the impact of the WHO Surgical Safety Checklist, as part of a raft of quality improvement measures, on national, regional and local patient outcomes. Attempts to introduce Checklists in resource poor settings have often relied on short-term visits by overseas agencies with limited scope for follow-up or ongoing support. Liaison with local governmental and non-governmental organisations, long-term volunteers and local leaders may represent a more sustainable model. While exact models are difficult to replicate, our experience is that a multi-agency approach may, where possible, be an effective one.

Haynes et al. [18] demonstrated that improvement in patient outcomes is associated with improved perceptions of teamwork and safety climates among workers in surgical departments who have introduced the WHO Surgical Safety Checklist [18]. It is notable that staff who were not present at the training day, but had been exposed to the Checklist through everyday use, were also highly enthusiastic about it. There remain issues, however, with the use of the Checklist at Yekatit 12 Hospital. The ‘Sign out’ section was clearly seen as more difficult, and less important, to complete than other sections. This is consistent with experience from the UK. [9] In addition, completion rates could be improved and the Checklist could be expanded to include other forms of anaesthesia, notably spinal anaesthesia.

The video and simulation sessions were reported as being useful aspects of our implementation strategy. Based on this feedback we have prepared an Ethiopian version of the ‘How to’ video, using our staff as actors, for use in other Ethiopian centres. We have also prepared a short documentary using interviews with key members of staff to advocate use of the WHO Surgical Safety Checklist in other Ethiopian hospitals. This video was presented at the 8th National Conference of the Ethiopian Anaesthetists’ Association 2012. Both videos are freely available via YouTube© (http://www.youtube.com/watch?v=tJ4NrJJrP0Q, http://www.youtube.com/watch?v=pFG9ihbPT-A) and have been incorporated into the ‘e-SAFE’ DVD, distributed by the United Kingdom’s Royal College of Anaesthetists, to aid anaesthetic education in low resource settings.

Checklists have great potential for reducing perioperative morbidity and mortality in both high and low resource settings, and the evidence base for their effectiveness is growing. However their effectiveness is dependent on their implementation and execution. We feel that the discussion between team members during the introduction of a Checklist, and the conversation around safety that this initiates, is of fundamental value to the quality improvement process. Implementation processes that impose Checklists autocratically, without consultation or discussion, may miss this essential component of team building and change to safety culture. We suspect that local ownership of the Checklist, and extension of it to other clinical areas, hinges around this early conversation. More work is required to delineate the features of successful quality improvement processes if Checklists are to realise their full potential in the effort to improve global surgical safety.

## Conclusion

Checklists have the potential to improve surgical safety, but their ability to do this hinges on the effectiveness of their implementation. A collaborative, multidisciplinary, approach using non-governmental organisations to support local leaders can be associated with high initial Checklist completion rates and staff satisfaction in a low-resource setting. Checklist adaptation is important both to suit local working practices but more importantly to engender a conversation around patient safety between different cadres of health professionals. Simulation and role play are useful tools during formal training, while supportive supervision following training may also be beneficial. Despite early success, Checklist compliance and completeness may decrease over time and strategies to explore and address this should be considered as part of any implementation.

## Competing interests

The authors declare that they have no competing interests.

## Authors’ contributions

TB coordinated the project, conducted the training, and performed supportive supervision and the one-month review. He wrote the original draft of this publication and is guarantor. SR performed and wrote the eight-month review. JM also acted as coordinator, performed supportive supervision and the one-month review. NHA devised teaching materials and the one-month review questionnaire. She also performed the eight-month review. ZM coordinated and provided support with teaching and with all levels of project design. YDG was the local lead for the project and was involved in consultation, Checklist adaptation, training, supervision and leadership. All contributors had roles in manuscript revision, have given final approval of the finished manuscript, and agree to be accountable for all aspects of the work.

## Supplementary Material

Additional file 1 One-month staff satisfaction questionnaire. Click here for file
