# Supplementary material for: Implementation of the WHO Surgical Safety Checklist in an Ethiopian Referral Hospital
Source: Patient Saf Surg. 2014 Mar 28;8:16. doi: 10.1186/1754-9493-8-16 (PMC4022152; doi:10.1186/1754-9493-8-16)
Supplement: Additional file 1 — One-month staff satisfaction questionnaire. [file 1754-9493-8-16-S1.pdf]

Yekatit 12 Hospital Plastic and Reconstructive Surgery Department

WHO Safe Surgery Checklist Staff Satisfaction Survey

This survey is anonymous

Please indicate your role in the department (check one box only)

- ☐ Nurse
- ☐ Anaesthetist
- ☐ Surgeon
- ☐ Health Officer
- ☐ Physician
- ☐ Resident
- ☐ Medical student
- ☐ Nursing student
- ☐ Other (please specify) ……

When did you start working in the department?

- ☐ Before 1st May 2012
- ☐ After 1st May 2012

Were you able to attend the WHO Safe Surgery Checklist training day?

- ☐ Yes
- ☐ No

This survey has 5 sections. The whole survey should take around 10 minutes to complete. Please mark answers with a tick (✓).

Section 1: Introduction of the WHO Safe Surgery Checklist

The Yekatit 12 WHO Safe Surgery Checklist was introduced on May 1st, 2012. Before it was introduced, there was discussion between surgeons, nurses and anaesthetists to make sure the checklist we used was appropriate. A training day was run involving a morning workshop and an afternoon session of simulation and role-play in the OR.

Please give your opinion on the following questions by marking the appropriate box:

	**Agree**	**Not sure**	**Disagree**
1. There was a need to improve patient safety before the checklist			
2. There was a need to improve communication before the checklist			
3. I was aware of the WHO Safe Surgery Checklist before it was introduced at Yekatit 12 Hospital			
4. I was able to give my opinion on the checklist before it was introduced			
5. The following aspects of the introduction were important:			
Informal discussion before the training day			
The staff meeting before the training day			
The morning workshop on the training day			
The videos on the training day			
The simulation session in the OR on the training day			
The role play in the OR on the training day			
Ongoing encouragement <u>after</u> the training day			
Filming the video in the OR after one month			
6. I am now confident to introduce using the WHO checklist to a colleague who is new to the department			
7. The introduction of the WHO Safe Surgical Checklist has been successful			

Section 2: Your opinion on the WHO Safe Surgery Checklist

We would like to know how you feel about the checklist and how it works.

Please give your opinion on the following questions by marking the appropriate box:

	Agree	Not sure	Disagree
1. Using the WHO Safe Surgery checklist is easy			
2. The WHO Safe Surgery checklist is quick to complete			
3. Using the checklist improves staff communication			
4. Using the checklist improves patient safety			
5. Using the checklist decreases the number of cancellations			
6. Using the checklist improves patient care			
7. Using the checklist allows equipment problems to be addressed			
8. Using the checklist has improved relationships between staff			

Section 3: The WHO Safe Surgery Checklist after one month

One month after introduction, we have found 82.6% (38/46) of general anaesthesia cases have a complete WHO Safe Surgery checklist. Of the 38 checklists completed, 15.7% (6/38) are not fully completed. All of the partially completed forms are missing a completed 'Sign out' section.

Please give your opinion on the following questions by marking the appropriate box:

	Agree	Not sure	Disagree
1. 82.5% is an acceptable percentage of completed forms			
2. In future the checklist should be used for (tick all that apply):			
General anaesthesia cases			
Spinal anaesthesia cases			
Sedation / local anaesthesia cases			
Outpatient cases			
ALL surgical cases, regardless of procedure or anaesthesia			
3. I think the most **difficult** part of the checklist to complete is: (tick **one** box only)			
Sign in (before induction of anaesthesia)			
Time out (before skin incision)			
Sign out (before patient leaves operating room)			
Specimen labelling (specimen / scrub room)			
4. I think the most **important** part of the checklist to complete is: (tick **one** box only)			
Sign in (before induction of anaesthesia)			
Time out (before skin incision)			
Sign out (before patient leaves operating room)			
Specimen labelling (specimen / scrub room)			
5. I think the WHO Safe Surgery checklist is working well for our department			
6. I think the WHO Safe Surgery checklist could be improved for our department			

If you answered ‘Agree’ to question 6, please give your ideas about how the checklist could be improved:

Section 4: Expanding the WHO Safe Surgery Checklist

The Ethiopian Federal Ministry of Health wants to use the WHO Safe Surgery Checklist for all hospitals in Ethiopia. The Regional Health Bureaus will be responsible for implementing this. We would like to use our experience at Yekatit 12 Hospital to encourage other hospitals to use the checklist.

Have you ever worked in a hospital, clinic or health centre other than Yekatit 12 Plastic and Reconstructive Surgery Department (<u>including</u> student placements)?

- ☐ Yes
- ☐ No

Please give your opinion on the following questions by marking the appropriate box:

	Agree	Not sure	Disagree
1. The WHO Safe Surgery Checklist would be applicable to the following types of centre (tick all that apply):			
Federal hospital			
University teaching hospital			
Referral or Specialised Hospital			
General Hospital			
District Hospital			
Health Centre			
Mission Hospital			
Private Hospital			
Private Higher Clinic			
2. The model of introduction used at Yekatit 12 Hospital Plastic and Reconstructive Surgery Department would be applicable to the following types of centre (tick all that apply):			
Federal hospital			
University teaching hospital			
Referral or Specialised Hospital			
General Hospital			
District Hospital			
Health Centre			
Mission Hospital			
Private Hospital			
Private Higher Clinic			
3. I would recommend using the WHO Safe Surgery Checklist to a colleague at another hospital			
4. If I had to have an operation, I would like the OR staff to be using the WHO Safe Surgery Checklist			

Section 5: Any other comments

Please tell us anything else you would like to about your experience with the WHO Safe Surgery Checklist at Yekatit 12 Hospital.

This is the end of the survey.

Thank you for your time.
